# An Unusual Case of Lithium-Induced Nephrogenic Diabetes Insipidus

**DOI:** 10.7759/cureus.100514

**Published:** 2025-12-31

**Authors:** Venkateshwara Reddy Malreddy, Laxmi M Balmuri

**Affiliations:** 1 Internal Medicine, Manchester Royal Infirmary, Manchester, GBR; 2 Diabetes and Endocrinology, Manchester Royal Infirmary, Manchester, GBR

**Keywords:** hypernatraemia, hypernatremia polyuria diabetes insipidus, hyperosmolar hyperglycemic state (hhs), lithium induced diabetes insipidus, nephrogenic diabetes insipidus

## Abstract

We report a case of a 53-year-old woman with bipolar affective disorder on long-term lithium therapy who presented with confusion, acute right lower quadrant abdominal pain, fever, vomiting, and increased urinary frequency, with suspected urinary sepsis. Initial assessment revealed very high C-reactive protein (CRP), severe hyperglycemia, and acute kidney injury (AKI Stage 3). She was treated for urinary sepsis as computed tomography (CT) imaging demonstrated bilateral pyelonephritis, and *Escherichia coli* was isolated from blood cultures. Lithium was withheld initially due to renal impairment.

As she met diagnostic criteria for hyperosmolar hyperglycemic state (HHS), she was treated as per the hospital hyperosmolar hyperglycemic state protocol. Glycemic control improved with appropriate fluid resuscitation and insulin administration. When renal function improved, lithium was restarted due to behavioral deterioration, following which she developed polyuria, severe hypernatremia, and persistently elevated serum osmolality. Due to severe agitation, she required intensive care unit (ICU) support for clinical and biochemical monitoring. Despite the resolution of hyperglycemia, persistent polyuria and hypernatremia prompted suspicion of diabetes insipidus (DI). Elevated serum sodium, high serum osmolality, and low urine osmolality were consistent with the diagnosis of DI. Normal brain imaging, elevated baseline co-peptin, and no response to desmopressin excluded central diabetes insipidus (CDI) and supported nephrogenic diabetes insipidus (NDI) secondary to chronic lithium use. She was treated with thiazide diuretics, with significant improvement in her urine output and stabilization of serum sodium. Lithium was discontinued, and referral to psychiatry was arranged.

This case highlights the diagnostic challenge in differentiating osmotic diuresis of HHS from lithium-induced NDI and emphasizes the need for clinical suspicion of NDI in patients on lithium therapy presenting with polyuria and electrolyte disturbances.

## Introduction

Hyperosmolar hyperglycemic state (HHS) and nephrogenic diabetes insipidus (NDI) are both characterized by profound hyperosmolality, hypernatremia, and polyuria, but they differ in underlying mechanisms and management [1,2]. HHS results from relative insulin deficiency with relative absence of ketosis, leading to osmotic diuresis from hyperglycemia. NDI, by contrast, results from renal resistance to antidiuretic hormone (ADH) and consequent impairment of urinary concentration [2,3].

Lithium is an important drug used in the management of bipolar affective disorder but is associated with several renal adverse effects, including nephrogenic diabetes insipidus (NDI), chronic kidney disease (CKD), and rarely, nephrotic syndrome [4,5]. NDI occurs in up to 40% of patients on long-term lithium therapy due to its inhibitory effects on vasopressin-mediated water reabsorption in the collecting ducts [6].

We present a rare case of lithium-induced NDI unmasked during the treatment of sepsis and HHS.

## Case presentation

A 53-year-old woman with a background of bipolar affective disorder on long-term lithium therapy presented to the emergency department with a three-day history of fever, vomiting, right lower quadrant pain, and increased urinary frequency. On arrival, she was confused (Glasgow Coma Scale 13/15), febrile (38.6 °C), tachycardic (112 beats/min), and hypotensive (86/54 mmHg).

Laboratory results are summarized in Table 1.

**Table 1:** Laboratory results on admission with reference values. mg/L: milligram per litre; µmol/L: micromole per litre; mL/min/1.73 m²: millilitre per minute per 1.73 metre square; mmol/L: millimole per litre; mOsm/kg: milliosmoles per kilogram.

Parameters	Result	Reference Range	Units
C-reactive protein (CRP)	300	0-5	mg/L
Serum creatinine	152	45-84	µmol/L
Estimated glomerular filtration rate (eGFR)	32	>90	mL/min/1.73 m²
Blood glucose	26	3.6-5.3	mmol/L
Serum ketones	Negative	-	-
Serum sodium	155	133-146	mmol/L
Serum potassium	3.7	3.5-5.3	mmol/L
Serum osmolality	345	275-295	mOsm/kg

Urinalysis showed pyuria and bacteriuria. CT imaging showed bilateral pyelonephritis, and *Escherichia coli *was isolated from blood cultures. A diagnosis of severe sepsis with acute kidney injury (AKI) and hyperosmolar hyperglycemic state (HHS) was made [1]. The patient met the HHS diagnostic criteria, which consist of marked hyperglycemia without significant ketonemia or acidosis(pH-7.38 and HCO3-21.4), severe dehydration, and measured plasma osmolality equal to or greater than 320 mOsm/kg [1].

Lithium was withheld on admission due to renal impairment. She was managed with intravenous antibiotics, aggressive fluid resuscitation, and, as per hospital protocol for HHS, including insulin infusion and close electrolyte monitoring under the guidance of the diabetes and endocrinology team. Renal function and blood glucose improved markedly over several days.

**Figure 1:**
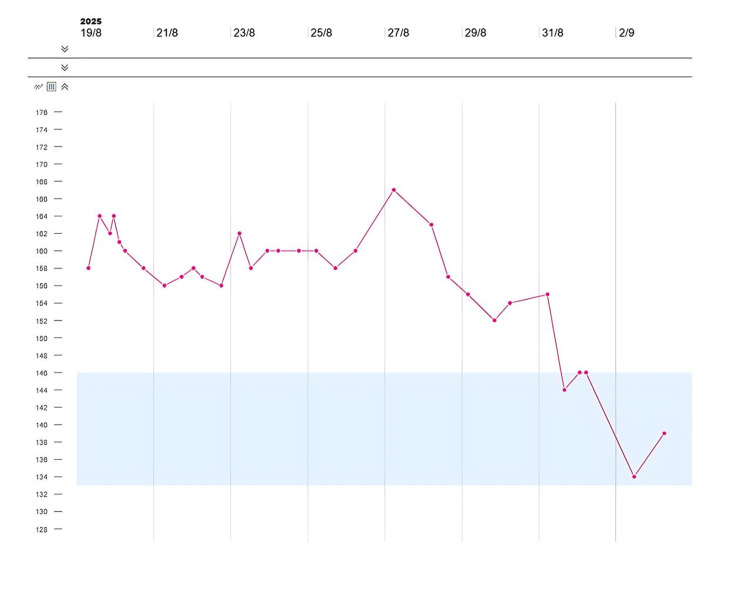
Timeline showing serum sodium (in millimoles per liter).

When renal function improved (serum creatinine 86 µmol/L), lithium was reintroduced because of behavioral concerns. Within 24 hours of restarting lithium, she became increasingly agitated, developed marked polyuria (>4L/day) and hypernatremia (164mmol/L) (Figure 1) despite adequate fluid replacement.

**Figure 2:**
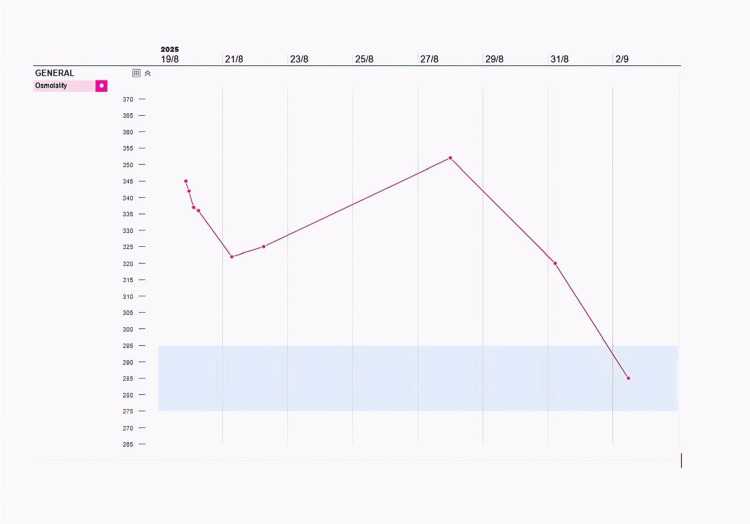
Timeline showing serum osmolality (in milliosmoles per kilogram).

Serum osmolality remained elevated (342 mOsm/kg) (Figure 2), requiring ICU admission for monitoring.

Persistent hypernatremia and polyuria following resolution of hyperglycemia and sepsis raised suspicion of diabetes insipidus. Head CT was unremarkable. A desmopressin challenge test produced no change in urine osmolality. Serum co-peptin was elevated to 18.6pmol/L (normal range 1-13.8 pmol/L), consistent with nephrogenic diabetes insipidus [2,3,7].

Lithium was discontinued, and thiazide diuretics commenced along with a low-sodium diet under endocrinology supervision. A psychiatric review was arranged for the alternative management of her bipolar disorder. Her serum sodium and osmolality gradually normalized (Figures 1, 2), and her polyuria resolved.

## Discussion

This case illustrates the diagnostic and therapeutic challenge when multiple osmotic and renal pathophysiological processes coexist. Hyperosmolar hyperglycaemic state (HHS) and nephrogenic diabetes insipidus (NDI) can present with overlapping biochemical features such as polyuria, hypernatremia, and elevated serum osmolality, but differ in aetiology [1,2].

**Figure 3:**
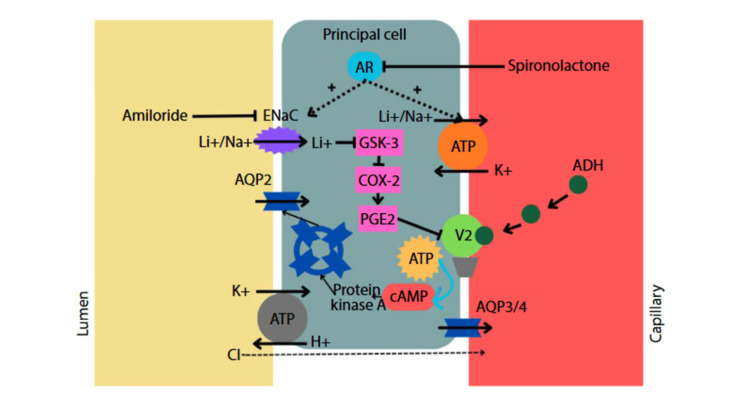
Pathophysiology of lithium-induced nephrogenic diabetes insipidus in the principal cell of the collecting duct. AR: aldosterone receptor; ENaC: epithelial sodium channel;  Li+: lithium;  Na+: sodium; GSK-3: glycogen  synthase  kinase  3; ATP: adenosine  triphosphate; ADH: anti-diuretic hormone; COX-2: cyclo-oxygenase 2; PGE2: prostaglandin E2; AQP: aquaporin; V2: vasopressin 2 receptor; K+: potassium; cAMP: cyclic adenosine monophosphate; H+: hydrogen ion; Cl-: chloride.

HHS results from osmotic diuresis due to hyperglycaemia, leading to water loss and hypernatremia. Standard management includes cautious fluid resuscitation, insulin infusion, and correction of electrolyte abnormalities [1]. Whereas NDI is due to renal resistance to antidiuretic hormone (ADH). This can be seen with lithium therapy in psychiatric patients [3]. Lithium-induced NDI is due to lithium entering the collecting-duct principal cells via epithelial sodium channels (ENaC), inhibiting cyclic adenosine monophosphate (cAMP) signalling and reducing aquaporin-2 expression, thereby impairing water reabsorption (Figure 3) [6,8-11].

In this patient, lithium-induced NDI was likely unmasked during treatment for sepsis and HHS. Reintroduction of lithium following renal recovery precipitated severe hypernatremia, suggesting pre-existing but compensated NDI [4,5]. Management of lithium-induced NDI includes discontinuation of lithium, fluid and electrolyte correction, and pharmacological measures such as thiazide diuretics or amiloride to reduce urine output. Thiazides cause mild volume depletion, enhancing proximal water reabsorption, while amiloride blocks lithium entry into tubular cells and may reduce nephrotoxicity [3,12].

This case emphasizes the importance of having a high index of suspicion for NDI in patients with polyuria and hypernatremia, particularly in those with a history of chronic lithium use, to avoid severe fluid and electrolyte imbalance and associated neurological complications.

## Conclusions

Lithium-induced nephrogenic diabetes insipidus is a rare but important differential diagnosis in patients with persistent hypernatremia and polyuria. Awareness of this overlap between hyperosmolar conditions is crucial, as management strategies differ significantly. Clinicians should maintain strict vigilance in patients on long term lithium therapy, and lithium should be discontinued promptly if NDI is suspected. Multidisciplinary management involving endocrinologists, nephrologists and psychiatrists is essential to improve patient outcomes.

## References

[1] Joint British Diabetes Societies for Inpatient Care (JBDS) Group (2022). The Management of Hyperosmolar Hyperglycaemic State (HHS) in Adults. The Management of Hyperosmolar Hyperglycaemic State (HHS) in Adults.

[2] Hui C, Khan M, Khan Suheb MZ (2025). Arginine Vasopressin Disorder (Diabetes Insipidus). [Updated 2024 Jan 11] In.. https://www.ncbi.nlm.nih.gov/books/NBK470458/.

[3] Bedford JJ, Weggery S, Ellis G, McDonald FJ, Joyce PR, Leader JP, Walker RJ (2008). Lithium-induced nephrogenic diabetes insipidus: renal effects of amiloride. Clin J Am Soc Nephrol.

[4] Boton R, Gaviria M, Batlle DC (1987). Prevalence, pathogenesis, and treatment of renal dysfunction associated with chronic lithium therapy. Am J Kidney Dis.

[5] Grünfeld JP, Rossier BC (2009). Lithium nephrotoxicity revisited. Nat Rev Nephrol.

[6] Kwon TH, Frøkiær J, Nielsen S (2013). Regulation of aquaporin-2 in the kidney: a molecular mechanism of body-water homeostasis. Kidney Res Clin Pract.

[7] Liu M, Deng M, Luo Q (2023). Metabolic reprogramming of renal epithelial cells contributes to lithium-induced nephrogenic diabetes insipidus. Biochim Biophys Acta Mol Basis Dis.

[8] Marples D, Frøkiaer J, Dørup J, Knepper MA, Nielsen S (1996). Hypokalemia-induced downregulation of aquaporin-2 water channel expression in rat kidney medulla and cortex. J Clin Invest.

[9] Mak A, Sung CC, Pisitkun T, Khositseth S, Knepper MA (2024). 'Aquaporin-omics': mechanisms of aquaporin-2 loss in polyuric disorders. J Physiol.

[10] Trepiccione F, Christensen BM (2010). Lithium-induced nephrogenic diabetes insipidus: new clinical and experimental findings. J Nephrol.

[11] Tatz SG, Blockman M, Dave AJ, Ross LI (2025). Severe lithium-induced nephrogenic diabetes insipidus: the diuresis paradox. S Afr Med.

[12] Şenocak Taşçi E, Eralp H, Kayataş K (2019). Lithium-induced nephrogenic diabetes insipidus: case report and review. Acta Endocrinol (Buchar).

